# Reply to Mekary, R.A.; Ding, E.L. Isotemporal Substitution as the Gold Standard Model for Physical Activity Epidemiology: Why It Is the Most Appropriate for Activity Time Research. *Int. J. Environ. Res. Public Health* 2019, *16*, 797

**DOI:** 10.3390/ijerph16162885

**Published:** 2019-08-12

**Authors:** Gregory J. H. Biddle, Charlotte L. Edwardson, Joseph Henson, Alex V. Rowlands, Thomas Yates

**Affiliations:** 1 Diabetes Research Centre, University of Leicester, Leicester LE5 4PW, UK; 2 NIHR Leicester Biomedical Research Centre, Leicester LE5 4PW, UK; 3 Health Sciences, University of Leicester, Leicester LE1 7RH, UK

Firstly, we would like to thank the authors for taking the time to review and comment on our paper. We believe it is invaluable to have open debates regarding current methodologies and the inference of research outputs. In the spirit of open debate and constructive discussion, we wish to respond to the observations of Mekary and Ding.

First, Mekary and Ding discuss the use of relative values within compositional data analysis (CODA), as opposed to the use of absolute values within a traditional isotemporal substitution model (ISM). Mekary and Ding recommend against the use of relative amounts in physical activity epidemiology, as physical activity guidelines are given in absolute amounts. We assert that in the context of a bounded day (24 h), what Mekary and Ding refer to as relative or absolute concepts actually amounts to the same thing. For example, 30 min per day will always be 2.1% of a day. A true absolute amount would not have the per day suffix. In neither case are we arguing that it would be sensible to use a non-fixed metric (i.e., % discretionary time) as the base measure. We postulate that the more interesting question is what unit of measurement is more appealing, minutes per day, or % of a day. Here, we believe in evidence-based guidelines, not guideline-based evidence. This approach allows guidelines to be altered and improved as the evidence grows. A good example of this is the development of 24 h guidelines for children aged 0–5 years in Canada [1]. Guidelines must always be adaptable to new evidence and must not hamper scientific progress. It may well be that in the future, guidelines will be based on the composition of physical behaviours conducted over a 24 h cycle, rather than different guidelines for different behaviours.

Second, Mekary and Ding debate the asymmetric results presented previously [2]. As stated, the compositional isotemporal model showed asymmetric results depending on whether time was reallocated to or from stepping time. For example, reallocating 30 min from sitting to stepping was associated with a 1.10-fold difference in Matsuda Insulin Sensitivity Index (Matsuda-ISI), whereas the reverse reallocation (30 min of stepping to sitting) was associated with a 0.87-fold difference. Importantly, we emphasised that these findings do not imply one model is in some way superior. We believe it is important to highlight the convergence and divergence in results, allowing for easier interpretation of previous results and aiding in the development of future analyses. Furthermore, this paper is not the first to highlight the asymmetry in compositional isotemporal results. A growing body of evidence suggests that the direction of each reallocation may impact on the magnitude of the observed association [3,4,5,6]. To the best of our knowledge, we believe that any cross-sectional linear ISM analysis that is based on non-transformed data, or where the dependant variable is log transformed (the most common methods employed in the literature) will always result in symmetrical findings, as it is inherent in the model. For example, using non-transformed normally distributed data, a coefficient for moving from sitting to walking of 2, will reverse to −2 when moving from walking to sitting, all other covariates being equal. This also applies to log transformed data, where a back transformed 2 fold difference in one direction will convert to its inverse (0.5) in the other. Whether this is a strength or weakness of one model over another has not been defined and will be based on observations from experimental research. 

Third, we agree with Mekary and Ding that the results between the CODA and ISM models were broadly similar; however, we are more positive regarding the potential for interpreting CODA analysis. The findings presented in this paper are supported by previous publications utilising a CODA approach [3,4,5,6,7,8,9,10,11], which allows for a greater understanding of the intrinsic codependence of these physical behaviours. This is not to say CODA provides a flawless interpretation of the associations of time spent in a variety of physical behaviours. Future work is required to improve the interpretability of the results from CODA analyses. 

To conclude, we believe our paper contributes to the growing evidence surrounding the associations of physical activity, sedentary behaviour and sleep on metabolic health. We refute the idea that our paper suggests superiority of CODA in comparison to ISM; however, we do stand by the appropriateness of CODA analyses in physical activity epidemiology and suggest future work is needed to improve the interpretability of these results. Ultimately, CODA is a developing methodological approach, one we consider important to nurture and develop. It provides a real solution for co-linearity between physical behaviours, allowing analyses to assess the associations of one behaviour, correctly adjusting for time spent in another. This reflects the true nature of behaviour, and for that reason, we believe it is worth pursuing. 

## References

[1] Tremblay M.S., Chaput J.P., Adamo K.B., Aubert S., Barnes J.D., Choquette L., Duggan M., Faulkner G., Goldfield G.S., Gray C.E. (2017). Canadian 24-Hour Movement Guidelines for the Early Years (0–4 years): An Integration of Physical Activity, Sedentary Behaviour, and Sleep. BMC Public Health.

[2] Biddle G., Edwardson C., Henson J., Davies M., Khunti K., Rowlands A., Yates T. (2018). Associations of Physical Behaviours and Behavioural Reallocations with Markers of Metabolic Health: A Compositional Data Analysis. Int. J. Environ. Res. Public Health.

[3] Chastin S.F.M., Palarea-Albaladejo J., Dontje M.L., Skelton D.A. (2015). Combined Effects of Time Spent in Physical Activity, Sedentary Behaviors and Sleep on Obesity and Cardio-Metabolic Health Markers: A Novel Compositional Data Analysis Approach. PLoS ONE.

[4] Dumuid D., Pedisic Z., Stanford T.E., Martin-Fernadez J.-A., Hron K., Maher C.A., Lewis L.K., Olds T. (2019). The compositional isotemporal substitution model: A method for estimating changes in a health outcome for reallocation of time between sleep, physical activity and sedentary behaviour. Stat. Methods Med Res..

[5] Dumuid D., Stanford T.E., Pedisic Z., Maher C., Lewis L.K., Martin-Fernandez J.A., Katzmarzyk P.T., Chaput J.P., Fogelholm M., Standage M. (2018). Adiposity and the isotemporal substitution of physical activity, sedentary time and sleep among school-aged children: a compositional data analysis approach. BMC Public Health.

[6] Fairclough S.J., Dumuid D., Taylor S., Curry W., McGrane B., Stratton G., Maher C., Olds T. (2017). Fitness, fatness and the reallocation of time between children’s daily movement behaviours: an analysis of compositional data. Int. J. Behav. Nutr. Phys. Act..

[7] Carson V., Tremblay M.S., Chaput J.-P., Chastin S.F. (2016). Associations between sleep duration, sedentary time, physical activity, and health indicators among Canadian children and youth using compositional analyses. Appl. Physiol. Nutr. Metab..

[8] Carson V., Tremblay M.S., Chastin S.F.M. (2017). Cross-sectional associations between sleep duration, sedentary time, physical activity, and adiposity indicators among Canadian preschool-aged children using compositional analyses. BMC Public Health.

[9] Dumuid D., Maher C., Lewis L.K., Stanford T.E., Martin Fernandez J.A., Ratcliffe J., Katzmarzyk P.T., Barreira T.V., Chaput J.P., Fogelholm M. (2018). Human development index, children’s health-related quality of life and movement behaviors: a compositional data analysis. Qual. Life Res..

[10] Gupta N., Dumuid D., Korshoj M., Jorgensen M.B., Sogaard K., Holtermann A. (2018). Is Daily Movement Behaviors’ Composition Related to Blood Pressure in Working Adults. Med. Sci. Sports Exerc..

[11] Gupta N., Korshoj M., Dumuid D., Coenen P., Allesøe K., Holtermann A. (2019). Daily domain-specific time-use composition of physical behaviors and blood pressure. Int. J. Behav. Nutr. Phys. Act..

